# Changing epidemiologic patterns of deliberate self poisoning in a rural district of Sri Lanka

**DOI:** 10.1186/1471-2458-12-593

**Published:** 2012-08-02

**Authors:** Lalith Senarathna, Shaluka F Jayamanna, Patrick J Kelly, Nick A Buckley, Michael J Dibley, Andrew H Dawson

**Affiliations:** 1 South Asian Clinical Toxicology Research Collaboration, Faculty of Medicine, University of Peradeniya, Peradeniya, Sri Lanka; 2 Sydney School of Public Health, University of Sydney, Sydney, NSW, Australia; 3 Department of Clinical Medicine, Faculty of Medicine, University of Kelaniya, Kelaniya, Sri Lanka; 4 Professorial Medicine Unit, POW Clinical School, University of New South Wales, Sydney, Australia; 5 Royal Prince Alfred Clinical School, University of Sydney, Sydney, NSW, Australia

## Abstract

**Background:**

Acute poisoning is a major public health issue in many parts of the world. The epidemiology and the mortality rate is higher in low and middle income countries, including Sri Lanka. The aim of this study was to provide details about the epidemiology of acute poisoning in a rural Sri Lankan district and to identify the changing patterns and epidemiology of poisoning.

**Methods:**

A prospective study was conducted from September 2008 to January 2010 in all hospitals with inpatient facilities in Anuradhapura district of North Central Province of Sri Lanka. Acute poisoning data was extracted from patient charts. Selected data were compared to the data collected from a 2005 study in 28 hospitals.

**Results:**

There were 3813 poisoned patients admitted to the hospitals in the Anuradhapura district over 17 months. The annual population incidence was 447 poisoning cases per 100,000 population. The total number of male and female patients was approximately similar, but the age distribution differed by gender. There was a very high incidence of poisoning in females aged 15–19, with an estimated cumulative incidence of 6% over these five years. Although, pesticides are still the most common type of poison, medicinal drug poisonings are now 21% of the total and have increased 1.6 fold since 2005.

**Conclusions:**

Acute poisoning remains a major public health problem in rural Sri Lanka and pesticide poisoning remains the most important poison. However, cases of medicinal drug poisoning have recently dramatically increased. Youth in these rural communities remain very vulnerable to acute poisoning and the problem is so common that school-based primary prevention programs may be worthwhile.

Lalith Senarathna, Shaluka F Jayamanna, Patrick J Kelly, Nick A Buckley,michael J Dibley, Andrew H Dawson. These authors contributed equally to this work.

## Background

Acute self poisoning is a major public health issue in many countries around the world. In developing countries such as Sri Lanka the reported mortality of 10% is significantly higher than the 0.5% reported in high income countries [1,2]. In Sri Lanka, acute poisoning is among the leading ten causes of hospital death [3,4]. The highest incidence is reported from rural districts. The high mortality reflects the wide availability of highly toxic compounds such as pesticides [5] and limited resources to treat poisoned patients in rural primary hospitals [1,6]. Recent studies in urban Sri Lanka demonstrate an increase in poisoning with pharmaceuticals [7]. The type of poisoning is influenced by availability as well as other factors such as prior knowledge about the poison and its effects gained through different means of communication and information including media [8]. More recent data highlighting longitudinal trends may have implications for health-care planning.

We aim to describe the epidemiology of poisoning in a rural district of Sri Lanka; the type of poisoning, the age and gender distributions of poisoning patients. We also examine the changing patterns of poisoning by comparing similar data collected from 28 of these hospitals in the same district in 2005 [9] with the corresponding subset from 2009.

## Methods

We prospectively collected 17 consecutive months of data on all admissions of poisoned patients in all hospitals in a rural district of Sri Lanka, including primary care peripheral hospitals and a secondary care hospital. The observational patient data collection in this study was conducted as a part of a cluster randomised controlled trial (ISRCTN73983810) of a brief educational intervention promoting poisoning treatment guidelines to hospital staff members. As this intervention was not directed to the community or patients it should not influence the incidence of poisoning. Data were collected for all patients admitted to all hospitals in Anuradhapura District of North Central Province of Sri Lanka, where poisoning has been in the top five causes of hospital deaths for the last 10 years. Anuradhapura district has a total population of 820,000 people (all ages) with 418,435 males and 401,567 females according to mid 2009 census data. This population is scattered in a land area of 7179 km^2^ (717900 hectare) with a population density of 114 persons per km^2^ (1.1 per hectare) [10]. The demographic details and socio-economic status of the population and health care service delivery of this rural district are similar to other rural districts of Sri Lanka [10].

There are 34 peripheral hospitals and one secondary care hospital, which serves as the referral centre for the study district. The peripheral hospitals operate as primary health care centres, and the first contact with the health care network. They all provide initial treatment for poisoned patients but transfer the majority of them to secondary care hospitals, which have medical intensive care units with specialised staff and better stocks of antidotes and medications.

### Data collection

The data collection from all consecutive poisoned patients admitted into all hospitals was started on September 2008 and continued up to January 2010. Details of all the patients who were 12 years of age or older, and who had a history of acute poisoning ingestion, were collected in this study. The patient records were compared to the admission log books in each hospital to ensure no patients were missed or wrongly diagnosed during data collection.

In peripheral hospitals, the staff members confirmed the exposure details of each patient on admission usually after viewing the poison product label/bottle, or seeing the remaining parts of the poison. In addition, family members and clinical symptoms were used to provide confirmation. Exposure, clinical assessment, treatment and outcome details were recorded in the patients’ case notes by the treating medical staff. Trained research assistants extracted these data from case records using a structured data collection form. The peripheral hospital patients who were transferred from peripheral hospitals to secondary care hospitals were followed up to record their hospital outcome. This was done by linking their outcome details with the peripheral hospital details by using simple algorithm which used hospital name, age, gender, date/time and poison type information. This linking also prevented double counting or repeat entry of individual patients. Coroners and police records were checked in the study district for any cases of out of hospital deaths from poisoning that may have occurred during the study period.

Data on patients who were admitted to the secondary hospitals in Anuradhapura district was collected as a part of an ongoing prospective observational cohort study [5]. This study collected the same data recorded in the peripheral hospitals plus additional clinical data from clinical examination. These details were prospectively entered in to this database by medically trained research assistants.

To assess changes in the patterns of poisons used and rates of admissions for poisoning, a comparison was made with data from a previous cross-sectional study conducted in 28 of these hospitals from July to December 2005 [9]. This study used the same methodology as the current study of peripheral hospitals. The difference was that in the 2005 study a retrospective review of admissions was undertaken for the previous 6months whereas in the subsequent peripheral hospital study the retrospective review was conducted on average every 3 weeks. In both studies retrieved notes were checked against the ward log of all admissions to ensure no cases were missed. The 2005 data was compared with data collected from the same hospitals during the same time of the year -July to December 2009. The same calendar months were used to minimise any variations in poison types during the year that might be related to seasonal variations in agricultural practises.

### Statistical analysis

Summary statistics were used to describe age, gender, and the types of poisons used by patients. 95% confidence intervals (CI) were calculated for differences in proportions where indicated. Chi-squared tests were used to compare between the proportions of patients according to the poison types ingested. The estimated mid-year population for 2009 in Anuradhapura district [10] was used to calculate the population incidences (poisoning admissions/population by age group/sex and poison).

## Ethical approval

This study protocol was reviewed and approved by the Human Research Ethics Committee of University of Sydney, Australia (Ref Number: 12083) and the Ethics Review Committee of University of Peradeniya, Sri Lanka.

## Results

### Epidemiology of poisoning

There were 3813 adult poisoned patients (above 12 years of age) admitted to all hospitals in Anuradhapura district of North Central Province of Sri Lanka during the 17 month data collection period. 3111 of these were first admitted to peripheral hospitals; 2287 (73.5%) patients were then transferred to the secondary care hospital for further treatment, 808 (26.0%) patients were discharged after treatment or left against medical advice, and 16 (0.5%) patients died in the peripheral hospital.

These poisoned patients ranged in age from 12 to 86 years with a median age of 24 years (IQR 19 to 35), and 1951 (51.2%) were females. The annual population incidence for the adult population (above age 15 years) in this area was 447/100,000 while the population incidence for females in the same age group was slightly higher than males (458 Vs 430/100,000). For the age group of 12 – 14 years, the estimated annual incidence was 101.5/100,000 for males and 246.2/100,000 for females.

### Age and gender variation

There were 21 patient records found without age and excluded from age related comparisons. This group had similar characteristics to the rest of the study population. Overall, patients were young, with 30% in 12 to 20 years range of age and over 50% less than or equal to 30 years of age (Table 1). The total numbers of male and female poisoning admissions were approximately similar, but the age distribution differed greatly between genders (Table 1). There were many more young female patients. In contrast, there was a larger proportion of middle aged and older males.

**Table 1:** The age distribution of male and female poisoned patients admitted September 2008 – January 2010 to hospitals in Anuradhapura District, Sri Lanka

**Age groups**	**Males n (%)**	**Females n (%)**
**12 - 19**	318 (17.1)	782 (40.1)
**20 – 29**	626 (33.9)	738 (38.0)
**30 – 39**	357 (19.3)	250 (12.9)
**40 – 49**	306 (16.6)	110 (5.7 )
**50 - 59**	169 (9.1)	41 (2.1)
**60 & Above**	73 (4.0)	22 (1.1)
**Total**	**1849**	**1943**

**Figure 1:**
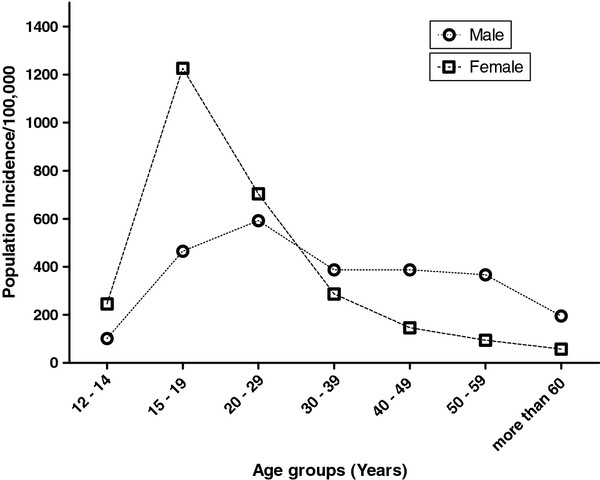
Estimated population incidence of acute poisoning for different age groups in Anuradhapura District of Sri Lanka – Calculated by using data from September 2008 to January 2010.

This is further demonstrated in the population incidence of male and female poisoning. The highest population incidence of 1226/100,000 was observed for females aged 15 to 19 years of age. Among this group of males it was 465/100,000. The incidence was higher in males than females over 30 (Figure 1), but declined in both sexes with increasing age.

### Types of poison

Pesticides including organophosphates, carbamate, herbicides and other unspecified pesticides were the most common agents used in poisoning in this rural district (1572 in total) (Table 2) and accounted for 41% (1572/3813) of the total admissions. This was followed by pharmaceuticals which accounted for 21% of all poisonings. Nearly half of these (45.4% (359/790)) were due to paracetamol. There was no other single pharmaceutical which featured prominently; the others were a wide range of prescribed drugs from antihypertensive, antipsychotic, anti-asthma and analgesic therapeutic classes. Yellow oleander (Thevetia peruviana) seeds were ingested by 392 (10.3%) patients**.**

**Table 2:** The types of pesticides ingested by self poisoned patients during September 2008 to January 2010 in Anuradhapura district, Sri Lanka

** Type of pesticide**	**n (% )**
**Carbamates**	
Carbosulfan	90 (5.8)
Carbofuran	49 (3.1)
Other carbamates	41 (2.6)
**Organophosphates**	
Chlorpyrifos	112 (7.1)
Dimethoate	90 (5.7)
Malathion	51 (3.2)
Profenophos	25 (1.6)
Other & unknown op	248 (15.8)
**Other pesticides**	
Paraquat	77 (4.9)
Glyphosate	231 (14.7)
MCPA^#^	114 (7.3)
Propanil	20 (1.3)
Bispyribac sodium	22 (1.4)
Fenoxaprop-p-ethyl	20 (1.3)
Other insecticides	178 (11.3)
Other herbicides	112 (7.1)
Unknown pesticide	92 (5.9)
Total	1572

#Methyl-4-Chlorophenoxyacetic acid.

The majority (84%) of patients who ingested medicines were less than 30 years of age and 69% of were females. For organophosphates, carbamate and paraquat, which are considered to be more toxic with higher mortality and morbidity, ingestion by males over twenty years old was higher than their female counterparts (Table 3).

**Table 3:** The demographics of patients admitted with self poisoning by type of poison. Data from Anuradhapura district of Sri Lanka - September 2008 to January 2010

**Age Groups**	**Organophosphate & Carbamate (n=704)**		**Paraquat (n=77)**		**Other Pesticides (n=783)**		**Medicine (n=785)**		**Oleander (n=392)**		**Other Poison (n=1051)**	
	**Male (%)**	**Female (%)**	**Male (%)**	**Female (%)**	**Male (%)**	**Female (%)**	**Male (%)**	**Female (%)**	**Male (%)**	**Female (%)**	**Male (%)**	**Female (%)**
**12 - 19**	7	11	7	10	8	17	7	37	16	24	8	18
**20 - 29**	18	15	33	13	19	16	9	32	19	18	17	17
**30 - 39**	16	8	10	1	12	6	2	7	6	7	10	6
**40 - 49**	11	3	16	1	11	3	1	3	5	2	10	3
**50 - 59**	7	2	5	1	5	1	1	1	3	1	6	1
**60 & Above**	3	1	1	1	2	0	0	1	0	0	3	1
**Total**	61	39	71	29	57	43	20	80	49	51	54	46

### Mortality rates

No deaths were identified to have occurred out of hospital. Within hospital 177 patients died during the study period and the over all mortality was 4.6%. The highest mortality was reported inmales 30 to 49 years of age and this group accounted for 32% (56 deaths) of the total deaths. Amongst females, the highest mortality was reported in the 20 – 29 year age group which had 29 deaths (16% of total deaths). Pesticides, including paraquat and organophosphates, caused most deaths (Table 4). Only 16 deaths occurred in peripheral hospitals, but there were 161 deaths in the secondary care hospital. However, 139 /161 secondary care hospital deaths were in patients transferred from peripheral hospitals. The population incidence in this district for all poisoning and pesticide poisoning death for people above 15 years of age was 21.5 and 11.6/100,000, respectively.

**Table 4:** Variation in case-fatality of poisoning according to demographic group and poison type. Data from Anuradhapura District of Sri Lanka - September 2008 to January 2010

	**Poisoned Patients (n)**	**Deaths (n)**	**Mortality (%)**
**Age Groups**			
12 - 19	1100	25	2.2
20 - 29	1364	56	4.1
30 - 39	607	35	6.7
40 - 49	416	28	5.8
50 - 59	210	16	7.6
60 & above	95	17	17.9
**Male**	1862	112	6.0
**Female**	1951	65	3.3
**Poison type**			
OP^*^& Carbamate	706	41	5.8
Paraquat	77	17	22.1
Other pesticides	789	38	4.8
Medicine	789	5	0.6
Oleander	393	16	4.1
Other & unknown poison^±^	1059	60	5.7
**Total**	3813	177	4.6

* Organophosphate.

± All other poison types including hydrocarbons, chemicals, plant substances and unknown poison types.

### Comparison of 2005 and 2009 data

There were 743 patients admitted to 28 peripheral hospitals during the 6months from July to December 2005 and 769 admitted to the same hospitals during the same months in 2009. There were only four and five deaths in these peripheral hospitals during 2005 and 2009 study periods respectively.

The median age and proportion of female patients were similar in 2005 and 2009. The types of poison ingested by patients changed from 2005 to 2009 (χ^2^=32.89, df=7, p=<0.001) (Table 5). In 2005, the number of patients with medicinal drug poisoning was low compared to pesticides and yellow oleander poisoning. Four years later medicine poisoning had increased 1.6 fold while poisoning with oleander seeds reduced (Table 5). Changes were most marked in those aged less than 30 years, particularly in the teenage group.

**Table 5:** Comparison of the type of poison used in self-poisoning in July- December 2005 and July-December 2009. Data from a subset of 28 hospitals in Anuradhapura district in Sri Lanka

	**In 2005**	**In 2009**	
**Type of poisoning**	**n (%)**	**n (%)**	**Difference of Proportions (95% CI)**
**Organophosphates**	138 (18.6)	116 (15.1)	−3.5 (−7.3 to 0.3)
**Carbamates**	36 (4.9)	35 (4.6)	−0.29 (−2.4 to 1.8)
**Other & Unknown pesticides**	196 (26.4)	177 (23.0)	−3.4 (−7.7 to 1.0)
**Paraquat**	18 (2.4)	18 (2.3)	−0.08 (−1. 6 to 1.5)
**Medicinal drugs**	83 (11.2)	138 (17.9)	6.8 (3.2 to 10.3)
**Oleander**	100 (13.5)	74 (9.6)	−3.8 (−7.16 to −0.6)
**Hydrocarbon**	54 (7.3)	38 (4.9)	−2.3 (−4.7 to 0.09)
**Other poison types**^**±**^	120 (16.2)	173 (22.5)	6.3 (2.4 to 10.3)
**Total**	743	769	

± All other poison types including hydrocarbons, chemicals, plant substances and unknown poison types.

## Discussion

This study reveals that the pattern of acute self poisoning is changing remarkably over just a few years in rural Sri Lankan districts with medicine (pharmaceutical) poisoning increasing rapidly. The population incidence of acute self poisoning was very high in this setting with young women being the most vulnerable. Although the crude country estimates of acute self poisoning appear to be similar across many low and middle income countries [11], there are important differences in the patterns; for example the type of poison, age and gender distribution and patient outcome. The type of poison (and consequently the lethality of deliberate self-poisoning) may vary according to accessibility of agents [11,12]. Most of the global death toll from pesticides is in the Asian region, where many people have ready access to pesticides [13].

Rural Sri Lanka shares this very high incidence of pesticide poisoning. The overall population incidence of self poisoning in the Anuradhapura district of Sri Lanka (447/100,000) appears to be higher than that reported from detailed surveys in other countries. For example, it is higher than reported rates from Oxford, UK (350/100,000) [14], Newcastle, Australia (266/100,000) [15], Mashhad, Iran (390/100,000) [16], and Oslo, Norway (200/100,000) [17].

Studies from both developed and developing countries demonstrate that young people, particularly women, below 30 years are over represented in self harm [18-23]. This trend is very evident in our study; teenage girls (12–19) accounted for 21% of all poisonings. The population incidence for females aged 15 to 19 years (1226/100,000) was approximately three times higher than that for males in this age group (465/100,000). This has also been observed in other studies; for example, in the United States of America (270.8 vs. 98.6/100,000) [21], Australia (375 vs. 159/100,000) [15], and Scotland (673 vs 365/100,000) [24]. Although female/male ratio in this study was similar to these other countries, the actual population incidence of poisoning of people aged 15 to 19 years in this area appears to be the highest reported.

The reasons for the observed higher population incidence in Sri Lanka were not within the scope of this study. Previous research in other settings in Sri Lanka [25-27] has suggested that self-poisoning is frequently impulsive [28,29] and not associated with psychiatric illnesses. Alcoholism, relationship and financial difficulties, family disputes, physical and psychological abuse were identified as the main reasons for attempting self poisoning [25,27]. These studies have not focused exclusively on young adults and adolescents who have self-poisoning. In the UK, this group indicated that feelings of loneliness, being unwanted, or anger were the main reasons and they used the act to alleviate or demonstrate the their distress [30,31].

The large increase in medicinal poisoning over a short period is the most remarkable aspect of the change in self poisoning behaviour in these communities. Increased awareness within the community through media reports or other means of communication has previously led to other rapid changes in the types of poisoning [8,32]. Common medicines such as paracetamol have been available in rural households for decades, provided as part of free health care in the country and available from local grocery shops without limitation [33]. Therefore availability alone does not seem sufficient reason for the recent changes in the use of these drugs in self poisoning.

There has been a compensatory decrease in oleander poisoning. As pharmaceutical poisonings have a much lower mortality (Table 4) the changing pattern of ingestion may reduce the harm from self-poisoning. However, these changes have implications when allocating treatment resources and developing health policy for treatment guidelines. For example most cases of paracetamol poisoning can be successfully treated in a peripheral hospitals with antidotes such as methionine or N- acetylcysteine thereby reducing expensive hospital transfers to secondary care hospitals [9]. Paracetamol poisoned patents who present within 10 hours of ingestion can be treated with methionine [34]. However, this requires the antidotes to be made available in these peripheral hospitals.

Government and public health authorities also need to include drug safety and community education components to the existing awareness programs. Other strategies, such as restricting package size, or limiting the availability of non-prescription medicines like paracetamol could be implemented to reduce the severity of acute poisoning.

### Limitations

The observational data collected on patients was collected in the context a cluster RCT of brief educational interventions to hospital staff members to promote adherence to poisoning treatment guidelines. As the RCT had no components directed to the community there would be no expected effect on the incidence of poisoning in the community. The effects of the educational intervention if successful on mortality would likely be small and not have a substantial impact on the estimates of mortality reported in this paper.

In the referral hospital data was only collected for patients who were 12 years or above. The population data from the Department of Statistics are only available in five year age groups and has population for 10 – 14 year age group. The lack of complete poisoning data from children aged 10 and 11 years meant we could not calculate an exact population incidence for the 10 – 14 years age group. As there was a high incidence in 15–19 year age group it was important to make an estimate of incidence in the 12–14 year age group as this would be valuable in planning the timing of delivery of public health interventions. As there is population data for the 10–14 year age groups, we assumed that these populations are evenly distributed in all ages and used an estimated population for the calculation of population incidence 12 to 14 year age group.

The incidence of pesticide poisoning can change in different quarters of the year due to the season-specific agricultural activities, which have a direct relationship with pesticide availability [6,12,28]. As all of these areas are irrigated this minimizes seasonal impact on variation in agriculture practices and by extension variation in pesticide use which is dictated by the type of agriculture. The predominant agriculture in this region is paddy rice and domestic vegetables. With the availability of water and a paddy growing season that lasts within 3–4 months, rice is grown year round. Therefore the data collection period to describe the epidemiology and pattern of poisoning should ideally be long enough not to be affected by short term seasonal variations. The 17 months data collection period used for this study should have minimized but not completely removed such potential biases.

However, a key strength of this study was that (unlike most previous studies in Sri Lanka) it described the epidemiology and patterns of poisoning in a complete district or a geographic patient catchment area that included all the hospitals in the area. The Sri Lankan public health care network is a well established and there is a hospital for every 3–4 villages. As we found no evidence of out of hospital deaths from poisoning in coroner or police records it seems likely that most severe cases present to hospital. It is possible that less severe poisonings may not present to hospital and that our population estimates for poisoning may be an underestimate.

The agricultural patterns, health care network and socio-economic status in this district are similar to other rural areas in the country. This is more evident due to the smaller size of the country – Sri Lanka is a small country with 65610 square kilometres. Therefore we believe the epidemiological data from this study is generalizable to other rural areas of the countr***y.*** And also, it is likely to be generalizable to other developing countries areas that are primarily agriculturally based. These data provided the opportunity to more accurately calculate the rural population incidence of poisoning with different substances and in different age and gender groups.

## Conclusions

Acute poisoning remains a major public health problem in rural Sri Lanka and pesticide poisoning remains the most important poison. However, cases of medicinal drug poisoning have recently dramatically increased. Youth in these rural communities remain very vulnerable to acute poisoning; The cumulative incidence of poisoning for females as they move through late adolescence appears on these figures to be around 6% (as repetition rates are very low [35]). This represents a very high risk group who should be targeted in primary prevention programs. A school based intervention to address the issues leading to self poisoning might even be effective in this setting.

## Competing interests

The authors declare that there are no competing interests.

## Authors’ contributions

LS designed this study, acted as principal researcher, collected and analysed data and wrote the first draft of the paper. SFJ participated in study design and data collection. PJK participated in data analysis and contributed to paper writing. NB participated in study design, data analysis and contributed to paper writing. MJD contributed in the study design, data analysis and paper writing. AHD also contributed to study design, data collection, data analysis and paper writing. All authors helped improve the study design and finalize paper writing. All authors read and approved the final manuscript.

## Pre-publication history

The pre-publication history for this paper can be accessed here:

http://www.biomedcentral.com/1471-2458/12/593/prepub

## References

[B1] Van der Hoek W Pesticide poisoning: amajor health problem in Sri Lanka Social Science &medicine 1998 46 4–5 495 504 9460829 10.1016/s0277-9536(97)00193-7

[B2] Bertolote JM Deaths from pesticide poisoning: a global response British Journal of Psychiatry 2006 189 201 203 10.1192/bjp.bp.105.020834 16946353 PMC2493385

[B3] Provincial Department of Health Services, Provincial Health Bulletin Provincial Department of Health Services 2006 North Central Province, Sri Lanka

[B4] Ministry of Health Sri Lanka Annual Heath Bulletin 2003 Ministry of Health, Sri Lanka

[B5] Dawson AH Acute human lethal toxicity of agricultural pesticides: a prospective cohort study PLoSmed 2010 7 10 e1000357 10.1371/journal.pmed.1000357 PMC2964340 21048990

[B6] Roberts DM Influence of pesticide regulation on acute poisoning deaths in Sri Lanka Bulletin of the World Health Organization 2003 81 11 789 798 14758405 PMC1693668

[B7] Silva DV Ratnayake A Increased use of medicinal drugs in self-harm in urban areas in Sri Lanka Arch Suicide Res 2008 12 4 366 369 10.1080/13811110802325265 18828040

[B8] Eddleston M Epidemic of self-poisoning with seeds of theyellow oleander tree (Thevetia peruviana) in northern Sri Lanka Tropical Medicine & International Health 1999 4 4 266 273 10.1046/j.1365-3156.1999.00397.x 10357862

[B9] Senarathna DLP How the level of resources and hospital staff attitude in primary care hospitals in rural Sri Lanka affect poisoning patient outcome in Centre for Clinical Epidemiology and Biostatistics 2006 University of Newcastle, Australia Newcastle

[B10] Department of Census and Statistics of Sri Lanka Statistics Handbook of Anuradhapura District 2010, [cited 2011 15th April ]; Available from: http://www.statistics.gov.lk/DistrictStatHBook.asp?District=Anuradhapura

[B11] Eddleston M Patterns and problems of deliberate self-poisoning in the developing world Qjm 2000 93 11 715 731 10.1093/qjmed/93.11.715 11077028

[B12] Mohamed F Pattern of pesticide storage before pesticide self-poisoning in rural Sri Lanka BMC Public Health 2009 9 10.1186/1471-2458-9-405 PMC2777873 19889236

[B13] Gunnell D The global distribution of fatal pesticide self-poisoning: systematic review BMC Public Health 2007 7 1 357 10.1186/1471-2458-7-357 18154668 PMC2262093

[B14] Hawton K Deliberate self-harm in Oxford, 1990–2000: a time of change in patient characteristics Psychological medicine 2003 33 06 987 995 10.1017/S0033291703007943 12946083

[B15] Reith DM Adolescent Self-Poisoning Crisis: The Journal of Crisis Intervention and Suicide Prevention 2003 24 2 79 84 10.1027//0227-5910.24.2.79 12880226

[B16] Afshari R Majdzadeh R Balali-Mood M Pattern of acute poisonings inmashhad, Iran 1993–2000 Journal of toxicology. Clinical toxicology 2004 42 7 965 975 15641642 10.1081/clt-200042550

[B17] Hovda K Acute poisonings treated in hospitals in Oslo: a one-year prospective study (I): pattern of poisoning Clinical Toxicology 2008 46 1 35 41 10.1080/15563650601185969 18167035

[B18] Chan Y A prospective epidemiological study of acute poisoning in Hong Kong Hong Kong J Emergmed 2005 12 3 156 161

[B19] Desalew M Pattern of acute adult poisoning at Tikur Anbessa specialized teaching hospital, a retrospective study, Ethiopia Human Exp Toxicol 2011 30 7 523 527 10.1177/0960327110377520 20630913

[B20] Pokhrel D A Comparative Retrospective Study of Poisoning Cases in Central, Zonal and District Hospitals Kathmandu University Journal of Science, Engineering and Technology 2010 4 1 40 48

[B21] Spiller HA Appana S Brock GN Epidemiological trends of suicide and attempted suicide by poisoning in the US: 2000–2008 Legal medicine 2010 12 4 177 183 10.1016/j.legalmed.2010.04.005 20547089

[B22] Srinivas Rao C Pesticide poisoning in south India: opportunities for prevention and improved medical management Tropical medicine & International Health 2005 10 6 p 581–8 10.1111/j.1365-3156.2005.01412.x PMC1762001 15941422

[B23] Zaidan ZAJ Deliberate self poisoning in Oman Tropical medicine & International Health 2002 7 6 549 556 10.1046/j.1365-3156.2002.00887.x 12031079

[B24] McLoone P Crombie IK Hospitalisation for deliberate self-poisoning in Scotland from 1981 to 1993: trends in rates and types of drugs used The British Journal of Psychiatry 1996 169 1 81 10.1192/bjp.169.1.81 8818373

[B25] Fernando R Study of suicides reported to the Coroner in Colombo, Sri Lanka Medicine, Science and the Law 2010 50 1 p 25 10.1258/msl.2009.009012 20349691

[B26] Hettiarachchi J Kodituwakku GC Pattern of Self poisoning in rural Sri Lanka:motivational aspects International Journal of Epidemiology 1989 18 2 418 22 10.1093/ije/18.2.418 2767856

[B27] Konradsen F Hoek W Peiris P Reaching for the bottle of pesticide–A cry for help Self-inflicted poisonings in Sri Lanka. Social Science &medicine 2006 62 7 1710 1719 10.1016/j.socscimed.2005.08.020 16165259

[B28] Eddleston M Choice of poison for intentional self-poisoning in rural Sri Lanka Clinical Toxicology: The Official Journal of the American Academy of Clinical Toxicology & European Association of Poisons Centres & Clinical Toxicologists 2006 44 3 283 286 10.1080/15563650600584444 PMC1940039 16749546

[B29] Hettiarachchi J Kodituwakku G Self poisoning in Sri Lanka:motivational aspects International Journal of Social Psychiatry 1989 35 2 204 10.1177/002076408903500209 2767925

[B30] Hawton K Motivational aspects of deliberate self-poisoning in adolescents The British Journal of Psychiatry 1982 141 3 286 10.1192/bjp.141.3.286 7139213

[B31] Hawton K Deliberate self harm in adolescents: a study of characteristics and trends in Oxford, 1990–2000 Journal of Child Psychology and Psychiatry 2003 44 8 1191 1198 10.1111/1469-7610.00200 14626459

[B32] Gawarammana IB Emerging epidemic of fatal human self-poisoning with a washing powder in Southern Sri Lanka: A prospective observational study Clinical Toxicology 2009 47 5 407 411 10.1080/15563650902915320 19492931 PMC3145130

[B33] Expert Committee on Essential medicine National List of Essential medicines 2009 Ministry of Health, Sri Lanka

[B34] Fernando R Management of poisoning 2007 National Poison Information Centre, National Hospital of Sri Lanka, State Printing Corporation, Sri Lanka

[B35] Mohamed F The prevalence of previous self-harm amongst self-poisoning patients in Sri Lanka Social Psychiatry and Psychiatric Epidemiology 2011 46 6 517 520 10.1007/s00127-010-0217-z 20372876 PMC3092923

